# Uncertainty and risk of misleading conclusions: an umbrella review of the quality of the evidence for ankle arthroscopy

**DOI:** 10.2340/17453674.2025.44330

**Published:** 2025-07-25

**Authors:** Ville PONKILAINEN, Valtteri PANULA, Juho LAAKSONEN, Anniina LAUREMA, Mikko MIETTINEN, Ville M MATTILA, Teemu KARJALAINEN

**Affiliations:** 1 Department of Orthopaedics and Traumatology, Tampere University Hospital; 2 Department of Surgery, Central Finland Central Hospital, Jyväskylä; 3 Department of Surgery, Mikkeli Central Hospital, Mikkeli; 4 Department of Orthopaedics and Traumatology, University of Helsinki and Helsinki University Hospital, Helsinki, Uusimaa; 5 COXA Hospital for Joint Replacement, Tampere; 6 Faculty of Medicine and Health Technology, University of Tampere, Tampere, Finland

## Abstract

**Background and purpose:**

Ankle arthroscopy is being increasingly utilized, but its potential benefits and harms remain unclear. This umbrella review aimed to assess the quality of systematic reviews and meta-analyses comparing ankle arthroscopy with equivalent open procedures or nonoperative options.

**Methods:**

A comprehensive search of MEDLINE, Embase, and CENTRAL was conducted on March 22, 2025. 2 reviewers independently screened abstracts and full texts, with conflicts resolved by a third reviewer. Systematic reviews assessing ankle arthroscopy versus any surgery or nonoperative treatment were included. The methodological quality of the reviews was evaluated using AMSTAR 2 criteria, along with an evaluation of whether the GRADE tool was appropriately applied.

**Results:**

The literature search identified 430 studies, of which 29 systematic reviews were included after the screening process. These reviews covered various conditions, including lateral ankle instability, osteoarthritis, fractures, and osteochondral defects. None of the systematic reviews included RCTs comparing arthroscopic procedures with nonoperative treatment. A methodological assessment using AMSTAR 2 criteria identified multiple critical flaws across all reviews, leading to an overall confidence rating of “critically low” for each. 1 study adequately applied the GRADE approach to assess the certainty of the evidence.

**Conclusion:**

The efficacy of ankle arthroscopic procedures remains based solely on observational evidence. Given the critically low methodological quality of existing reviews, conclusions suggesting benefits of ankle arthroscopy, particularly over open procedures, are unreliable and insufficient to inform clinical recommendations. RCTs comparing ankle arthroscopy with nonoperative treatments or sham surgery are urgently needed.

Ankle arthroscopy is a widely used technique for diagnosing and treating intra-articular conditions of the ankle joint. The rationale for ankle arthroscopy includes enhanced diagnostic precision and decreased soft-tissue trauma compared with open procedures [1]. Ankle arthroscopy has been used in treating chronic lateral ankle instability, ankle fractures, talar osteochondral defects, ankle osteoarthritis, impingements, removing loose bodies, septic arthritis, arthrofibrosis, and synovitis [2]. It has also been used in reducing joint surface fragments in intra-articular ankle fractures [3]. Multiple literature reviews have advocated the use of ankle arthroscopy and suggested that it is superior or comparable to open procedures in the Broström procedure [4], ankle arthrodesis [5,6], and arthroscopy-assisted ankle fracture surgery [3,7]. However, a recent study identified significant regional variation in its utilization, indicating that either the evidence is not sufficiently reliable to gain widespread acceptance or that robust evidence exists but has not been effectively disseminated [8,9].

To evaluate the efficacy of ankle arthroscopy, systematic reviews and meta-analyses are widely regarded as the main tools also for informing clinical guidelines and treatment recommendations. However, the reliability of a systematic review and meta-analysis depends largely on the quality of the original studies they include, and how the uncertainties of the evidence are reflected in the evidence synthesis. The GRADE approach [10] has highlighted that study design alone is insufficient to determine the certainty of evidence; rather, a structured assessment of risk of bias, inconsistency, imprecision, indirectness, and publication bias is necessary. Any flaws in the synthesis process can lead to biased treatment effect estimates and misleading conclusions. Therefore, for the analysis of ankle arthroscopy, it is crucial to conduct the analyses rigorously as well as communicate the uncertainties of evidence on which treatment decisions are based [10-14]. Concerns over the methodological flaws in orthopedic meta-analyses were raised as early as 2001 [15], and although research quality has generally improved since then, a substantial proportion of studies still exhibit major to extensive methodological shortcomings [16,17], including those published in top orthopedic journals [18].

This umbrella review aimed to evaluate the existing evidence on the efficacy of arthroscopy-assisted ankle procedures and determine whether the systematic reviews informing clinical recommendations are methodologically rigorous and their conclusions are supported by best evidence.

## Methods

### Study design

We included systematic reviews that followed the PICO framework [19]: Patients: adult patients with any ankle joint condition; Intervention: ankle joint arthroscopic procedures; Comparison: any surgery (including placebo surgery), non-surgical approaches, or open ankle joint procedures; Outcomes: Pain, global improvement, health-related quality of life, participation (return to work or leisure activities), and adverse events. We included all systematic reviews with or without meta-analysis that included observational (non-randomized) and randomized studies meeting the same PICO criteria as the original studies in the umbrella review. Reviews were classified as systematic if the authors explicitly identified them as systematic reviews.

The study was reported in accordance with the PRIOR statement [20].

### Search and data extraction

Searches were conducted in the MEDLINE, Embase, and CENTRAL databases on March 22, 2025 (Figure). Search strategies are included in Appendix 1. 2 reviewers independently screened the abstracts and full-text articles, with any conflicts resolved by a third author. Screening was conducted using Covidence software [21]. Additionally, the reference lists of the included articles were screened to identify additional relevant studies.

**Figure:**
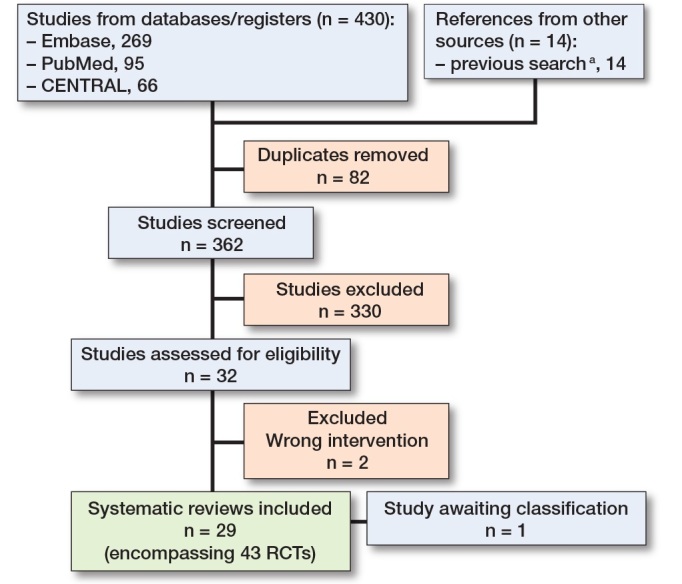
Review process as a flowchart. ^a^ Previous search strategy and flowchart is provided in Appendix 3 and 4 , see Supplementary data.

Data extraction was performed unblinded by 2 authors: 1 author extracted the data, while a second author reviewed it for inconsistencies. As the aim of this review was to assess the quality of the systematic reviews and whether their conclusions were sound, we did not perform a meta-analysis to quantify treatment effects of procedures. Data was reported using descriptive methods.

The following information was extracted from the studies: characteristics of the intervention and control group, the Risk of Bias (RoB) tool, if certainty of evidence was evaluated with the GRADE tool [10,22], primary outcome(s), primary timepoints, number of included studies, number of included RCTs, review conclusion, clinical recommendation, and the method used to assess the certainty of evidence. The number of included RCTs was confirmed by checking all the original references that the authors labeled as RCTs. In the assessment of certainty of evidence, a study was categorized as “Yes” if the certainty of evidence was evaluated based on the quality of the included studies, meaning that the GRADE approach was applied correctly. The use of GRADE was deemed appropriate when authors assessed the certainty of evidence for each outcome from “High” to “Very Low,” based on the domains of risk of bias, inconsistency, imprecision, indirectness, and publication bias [10]. A study was categorized as “Partially” if the conclusion mentioned that the results were based on retrospective studies or if additional research was deemed necessary. Finally, a study was categorized as “No” if the conclusion did not address these aspects. The assessment was based on the conclusions presented in the abstract and the final conclusions section.

### Quality assessment

The methodological quality of the reviews was assessed using A MeaSurement Tool to Assess systematic Reviews II (AMSTAR 2) criteria [23]. AMSTAR 2 was selected over other tools (e.g., ROBIS [24]) because our focus was exclusively on assessing the methodological quality of the included reviews. According to the original AMSTAR 2 publication, items 2, 4, 7, 9, 11, 13, and 15 were identified as critical domains [23]. Reviews were rated based on their methodological quality using 4 categories: High, Moderate, Low, and Critically Low. A review was classified as High quality if it had no or only 1 non-critical weakness, ensuring an accurate and comprehensive summary of the available studies. Moderate quality was assigned to reviews with more than 1 non-critical weakness but no critical flaws, meaning the summary of results was still likely to be accurate. Reviews with 1 critical flaw, regardless of additional non-critical weaknesses, were rated as Low quality, as they might not provide a reliable summary of the available evidence. Finally, reviews with more than 1 critical flaw were considered Critically Low quality, indicating that they should not be relied upon for an accurate and comprehensive synthesis of the studies [23].

### Protocol deviations

Initially, the aim of our review was to identify RCTs comparing ankle arthroscopy with placebo or sham surgery or nonoperative treatments. However, as no such RCTs were found, we amended our objective. Specifically, we chose to conduct an umbrella review, qualitatively assessing the quality of existing reviews using the AMSTAR II criteria. Because current guidelines and clinical practices rely heavily on these reviews, we aimed to assess their quality. Given the generally poor quality, the findings of these reviews are not suitable for clinical use. Due to these quality issues, quantitative synthesis is not feasible, as biased primary estimates would lead to biased pooled results. Therefore, we chose to refrain from conducting a post-hoc meta-analysis. This change was made early in the review process, allowing us to adapt our methodology accordingly and provide a broader overview of the current evidence base. These amendments were reported in the PROSPERO registration prospectively (https://www.crd.york.ac.uk/PROSPERO/view/CRD42024618073).

### Ethics, registration, funding, and disclosures

This work is a systematic review of published studies; no primary human or animal research was conducted. Therefore, institutional review board approval was not required. This systematic review was prospectively registered in PROSPERO: CRD42024618073 [25]. This research received no external funding. The datasets generated and analyzed during this study are available from the corresponding author upon request. The authors declare that they have no competing interests. Complete disclosure of interest forms according to ICMJE are available on the article page, doi: 10.2340/17453674.2025.44330

## Results

The literature search identified 430 studies (see Figure). After excluding 68 duplicates, title and abstract screening was conducted for 362 studies, resulting in 32 articles to full-text phase. Following full-text review, 30 studies were included, and 2 studies were excluded due to not fulfilling the inclusion criteria and 1 study [23] was classified as “awaiting classification,” as it was an abstract of a study that was not published (Appendix 2). 29 systematic reviews were included in this umbrella review, encompassing 457 original publications, of which 43 (9%) were RCTs [2-7,27-49].

The included studies investigated the effect of ankle arthroscopy for various conditions: lateral ankle instability (n = 13), ankle osteoarthritis (n = 6), ankle fractures (n = 6), osteochondral defects (n = 3). 1 review encompassed numerous indications, including all previously noted conditions as well as impingement, loose bodies, septic arthritis, arthrofibrosis, and synovitis [2]. In the included studies, meta-analysis was conducted in 19 of them. None of the systematic reviews included RCTs that compared ankle arthroscopy with nonoperative treatment, placebo, or sham surgery. None of the included reviews prespecified their primary timepoints.

### Arthroscopic reconstruction for ankle instability

14 systematic reviews investigated the effect of arthroscopic surgery for lateral ankle instability [2,4,42,30,32,36,38,39,41,42,45-49] comparing arthroscopic and open lateral ankle ligament repair procedures in an adult population. 10 reviews performed meta-analysis. The reviews were published between 2015 and 2024. They included a median of 8 (range 4–44) studies and median of 420 (range 207–2,041) patients. 9 reviews included RCTs, yet none of the reviews performed analyses using only RCTs. The primary outcomes were patient-reported outcome measures (PROMs) in all studies.

7 of the reviews did not assess the risk of bias of the included studies, while 4 used the RoB I or II tool. None of the studies employed the GRADE approach to assess the certainty of the evidence. Overall confidence rating based on AMSTAR 2 criteria was Critically Low for all reviews (flaws in 6–16 out of 16 criteria) assessing arthroscopic surgery for lateral ankle instability (Table 1). Flaws were present in 5–7 out of 7 critical items.

**Table 1:** AMSTAR 2 criteria for each review

Study	Items (see below)															
	1	2^a^	3	4^a^	5	6	7^a^	8	9 a	10	11^a^	12	13^a^	14	15^a^	16
**Arthroscopic reconstruction for ankle instability**																
Attia 2021 [4]	Yes	No	No	Partial	Yes	No	No	Partial	Partial	No	No	No	No	No	Yes	No
Alhaddad 2023 [41]	Yes	Partial	No	Partial	Yes	Yes	No	Yes	Partial	No	–	–	No	No	–	Yes
Brown 2018 [38]	No	No	No	Partial	Yes	Yes	No	Partial	No	No	–	–	No	No	–	Yes
Brown 2020 [42]	Yes	No	No	Partial	Yes	Yes	No	Partial	No	No	No	No	No	No	No	Yes
Guelfi 2016 [36]	Yes	No	No	No	Yes	No	No	Partial	No	No	No	No	No	Yes	No	Yes
Matsui 2016 [30]	No	No	No	Partial	No	No	No	No	No	No	–	–	No	No	No	Yes
Moorthy 2021 [32]	Yes	Partial	Yes	Partial	Yes	Yes	No	Partial	Partial	No	Yes	Yes	No	Yes	Yes	Yes
Song 2018 [39]	Yes	Partial	Yes	Partial	Yes	Yes	No	Yes	Partial	No	Yes	Yes	Yes	Yes	No	Yes
Tonsuthanluck 2024 [45]	Yes	Partial	Yes	Partial	Yes	Yes	No	Partial	Partial	No	No	No	No	No	No	Yes
Wang 2024 [46]	Yes	No	No	Partial	Yes	Yes	No	Partial	No	No	No	No	No	Yes	Yes	Yes
Wittig 2022 [47]	Yes	No	Yes	Partial	No	No	No	Yes	No	No	No	No	No	Yes	No	Yes
Zhao 2023 [48]	Yes	Partial	Yes	Partial	Yes	Yes	No	Yes	Partial	No	Yes	Yes	Yes	Yes	No	Yes
Zhi 2020 [49]	Yes	Partial	Yes	Partial	Yes	Yes	No	Yes	Partial	No	Yes	No	No	No	No	Yes
**Arthroscopic-assisted fusion for ankle osteoarthritis**																
Bai 2021 [5]	Yes	Partial	No	Partial	Yes	Yes	No	Partial	Partial	No	Yes	No	No	No	Yes	Yes
Honnenahalli 2017 [37]	Yes	Partial	Yes	Partial	Yes	Yes	No	Yes	Partial	No	Yes	No	Yes	Yes	Yes	Yes
Lorente 2023 [43]	Yes	Partial	Yes	Partial	Yes	Yes	Yes	Partial	Partial	No	Yes	Yes	Yes	Yes	Yes	Yes
Mok 2020 [31]	Yes	Partial	No	Partial	Yes	Yes	No	Yes	Partial	No	Yes	Yes	Yes	Yes	Yes	Yes
Park 2018 [40]	Yes	No	No	Partial	Yes	Yes	No	Yes	No	No	–	–	No	Yes	–	Yes
Xing 2023 [6]	Yes	Partial	No	Partial	No	No	No	Partial	Partial	No	No	No	Yes	No	Yes	Yes
**Arthroscopic-assisted reduction for ankle fractures**																
Chen 2015 [27]	No	No	No	No	Yes	Yes	No	Partial	No	No	–	–	No	No	–	Yes
Gonzalez 2016 [7]	No	No	No	No	No	No	No	No	No	No	–	–	No	No	–	Yes
Lee 2017 [3]	Yes	Partial	No	Partial	Yes	Yes	No	Partial	Partial	No	No	No	No	No	Yes	Yes
Meyer-Pries 2025 [44]	Yes	Partial	Yes	Partial	Yes	Yes	No	Partial	Partial	No	No	No	No	Yes	No	Yes
Zhang 2023 [34]	Yes	Partial	No	Partial	Yes	No	No	Partial	Partial	No	Yes	Yes	Yes	No	Yes	Yes
Zhuang 2023 [35]	Yes	Partial	No	Partial	Yes	Yes	No	Partial	Partial	No	Yes	No	No	Yes	Yes	No
**Debridement or autologous chondrocyte implantation for osteochondral lesions**																
Erickson 2018 [28]	Yes	No	No	Partial	Yes	Yes	No	No	No	No	–	–	No	No	–	Yes
Marin Fermin 2021 [29]	No	Partial	No	Partial	Yes	Yes	Yes	No	Partial	No	–	–	No	No	–	Yes
Zengerink 2010 [33]	No	No	No	Partial	Yes	No	No	No	Partial	No	–	–	No	No	–	No
**Multiple indications**																
Glazebrook 2009 [2]	No	No	No	No	Yes	No	No	No	No	No	–	–	No	No	–	No
Item 1	Did the research questions and inclusion criteria for the review include the components of PICO?															
Item 2	Did the report of the review contain an explicit statement that the review methods were established prior to the conduct of the review and did the report justify any significant deviations from the protocol?															
Item 3	Did the review authors explain their selection of the study designs for inclusion in the review?															
Item 4	Did the review authors use a comprehensive literature search strategy?															
Item 5	Did the review authors perform study selection in duplicate?															
Item 6	Did the review authors perform data extraction in duplicate?															
Item 7	Did the review authors provide a list of excluded studies and justify the exclusions?															
Item 8	Did the review authors describe the included studies in adequate detail?															
Item 9	Did the review authors use a satisfactory technique for assessing the risk of bias (RoB) in individual studies that were included in the review?															
Item 10	Did the review authors report on the sources of funding for the studies included in the review?															
Item 11	If meta-analysis was performed, did the review authors use appropriate methods for statistical combination of results?															
Item 12	If meta-analysis was performed, did the review authors assess the potential impact of RoB in individual studies on the results of the meta-analysis or other evidence synthesis?															
Item 13	Did the review authors account for RoB in primary studies when interpreting/discussing the results of the review?															
Item 14	Did the review authors provide a satisfactory explanation for, and discussion of, any heterogeneity observed in the results of the review?															
Item 15	If they performed quantitative synthesis, did the review authors carry out an adequate investigation of publication bias (small study bias) and discuss its likely impact on the results of the review?															
Item 16	Did the review authors report any potential sources of conflict of interest, including any funding they received for conducting the review?															

“–”: No meta-analysis conducted

a AMSTAR 2 critical domains.

4 reviews concluded that arthroscopic surgery is as good as open surgery [32,36,46,48], while 10 reviews concluded it is superior to open surgery [2,4,30,38,39,41,42,45,47,49], none acknowledging that the evidence was by and large drawn from observational studies and none performed analysis based on RCTs (Table 2). 1 review [36] claimed excellent efficacy despite none of the included studies directly assessing the procedure’s efficacy.

**Table 2:** Conclusions, clinical recommendations, and overall confidence of methodological quality of the included studies

Author	Conclusion	Clinical recommendation	Design of the included studies reflected in the conclusion	AMSTAR 2 criteria No. of Yes/16	AMSTAR 2 overall confidence	GRADE applied
**Arthroscopic reconstruction for ankle instability**						
Attia 2021	“Arthroscopic Broström is superior to open Broström-Gould surgery”	No recommendation	Partially	3	Critically low	No
Alhaddad 2023	“Although the arthroscopic technique had higher complication rates than the modified Brostrom technique, the difference was insignificant. Therefore, we concluded that surgeons performing the arthroscopic Brostrom technique should have good arthroscopic skills to minimize complications”	No recommendation	No	5	Critically low	No
Brown 2018	“Arthroscopic ankle arthrodesis has more advantages than open ankle arthrodesis”	No recommendation	No	3	Critically low	No
Brown 2020	“Short-term AOFAS functional outcome scores were significantly improved with arthroscopic lateral ankle repair compared to open repair. There was no significant difference between arthroscopic and open repair with regards to Karlsson functional outcome score, total complication rate, or the nerve and wound complication”	No recommendation	Partially	4	Critically low	No
Guelfi 2018	“The results of this review show the excellent efficacy of open and arthroscopic surgical procedures in the treatment of the chronic ankle instability”	No recommendation	Partially	4	Critically low	No
Matsui 2016	“There was currently poor quality of evidence that was insufficient to allow a high grade of recommendation to support the use of the MIS”	No recommendation	Partially	1	Critically low	No
Moorthy 2021	”Arthroscopic repairs offer comparable clinical outcomes with a lower wound complication rate, compared to traditional open repairs”	No recommendation	Partially	9	Critically low	No
Song 2018	“No statistically significant difference in outcome measures between arthroscopic versus open repair, both of which reported favorable and satisfactory outcomes, and produced equivalent clinical result”	No recommendation	Partially	10	Critically low	No
Tonsuthanluck 2024	“The findings support near-future developments validating arthroscopic repair as the new gold Standard for LLC repairs”	No recommendation	No	5	Critically low	No
Wang 2024	“The study findings suggested that keyhole surgery may be beneficial in patients with persistent lateral ankle joint instability”	No recommendation	Partially	6	Critically low	No
Wittig 2022	“Similar to open repair, all-arthroscopic ligament repair for chronic lateral ankle instability is a safe treatment option that yields excellent clinical outcomes”	No recommendation	No	5	Critically low	No
Zhao 2023	“Arthroscopic repair yields comparable outcomes to open surgery”	“We advocate for adopting arthroscopic repair as a preferred alternative to the conventional open Broström-Gould procedure”	No	10	Critically low	No
Zhi 2020	“Arthroscopic repair for lateral ankle instability shows excellent clinical results comparable to open repair. Especially, arthroscopic repair might alleviate more pain due to the minimally invasive procedure”	No recommendation	No	7	Critically low	No
**Arthroscopic-assisted fusion for ankle osteoarthritis**						
Bai 2021	“Arthroscopic ankle arthrodesis has more advantages than open ankle arthrodesis in improving the fusion rate and reducing complications”	“The overall clinical effect is better than that of open ankle fusion, and it is worthy of popularization and application”	No	6	Critically low	Partially
Honnenahalli 2017	“The best available evidence demonstrates that arthroscopic ankle fusion may be associated with a higher fusion rate, shorter tourniquet time, and shorter length of stay compared to open ankle fusion. We found no significant difference between two groups in terms of infection rate, overall complication rate, and operation time”	No recommendation	Partially	10	Critically low	No
Lorente 2023	“Our findings showed a non-statistically significant fusion rate. On the other hand, operation time was similar among both surgical techniques, without significant differences. Nevertheless, lower hospital stay was found in patients that were operated on with arthroscopy”	No recommendation	No	11	Critically low	Yes
Mok 2020	“Arthroscopic arthrodesis was associated with a higher fusion rate, smaller estimated blood loss, shorter tourniquet time, and shorter length of hospitalisation than open surgery”	No recommendation	Partially	10	Critically low	No
Park 2018	“AAA was shown to offer the advantages of better clinical scores, fewer complications, a shorter hospital stay, and less blood loss compared with OAA. However, the union rate, reoperation rate, and operation time were similar overall between the 2 groups”	No recommendation	No	6	Critically low	No
Xing 2023	“Arthroscopic ankle arthrodesis is superior to open ankle arthrodesis alone in the treatment of ankle arthritis”	No recommendation	Partially	4	Critically low	No
**Arthroscopic-assisted reduction for ankle fractures**						
Chen 2015	“Acute ankle fractures are commonly concomitant with multiple soft-tissue injuries in which arthroscopy may serve as a method for accurate diagnosis and appropriate treatment”	No recommendation	No	3	Critically low	No
Gonzalez 2016	“Ankle arthroscopy is a valuable tool in identifying and treating intra-articular lesions associated with ankle fractures”	No recommendation	No	1	Critically low	No
Lee 2017	“The arthroscopically assisted ORIF for ankle fractures were more beneficial than the conventional ORIF in the current evidences”	No recommendation	No	5	Critically low	No
Meyer-Pries 2025	“AORIF might improve postoperative outcomes without increasing the complication rate when compared to conventional ORIF”	No recommendation	Partially	6	Critically low	No
Zhang 2023	“ARIF and ORIF are comparable in terms of providing pain relief and improving function for patients with ankle fractures”	No recommendation	No	7	Critically low	No
Zhuang 2023	“ARIF for ankle fractures might be beneficial to offer superior functional outcomes and VAS score than ORIF”	No recommendation	No	6	Critically low	No
**Debridement or autologous chondrocyte implantation for osteochondral lesions**						
Erickson 2018	“No procedure demonstrates superiority or inferiority between the combination of open or arthroscopic MACI and PACI in the management of OLT less than 2.5 cm^2^”	No recommendation	Partially	4	Critically low	No
Marin Fermin 2021	“There is a paucity of evidence evaluating arthroscopic debridement alone for osteo-chondral lesion treatment in the last 2 decades. Bone-marrow stimulation techniques remain the first-line surgical strategy for OLT treatment without proven superiority”	No recommendation	No	4	Critically low	No
Zengerink 2010	“Treatment by means of debridement and bone marrow stimulation is the most effective treatment strategy for symptomatic osteo-chondral lesions of the talus”	No recommendation	Partially	1	Critically low	No
**Multiple indications**						
Glazebrook 2009	“There exists adequate evidence-based literature to support the surgical technique of ankle arthroscopy for most current generally accepted indications”	Grade B, C, or I recommendations per indication	Partially	1	Critically low	No

### Arthroscopic-assisted fusion for ankle osteoarthritis

7 reviews investigated the effect of arthroscopic surgery for ankle osteoarthritis [2,5,6,31,37,40,43] comparing arthroscopic and open ankle arthrodesis in adult population. 5 reviews performed meta-analysis. The reviews were published between 2015 and 2023. They included a median of 10 studies (range 6–18) and median of 487 (range 286–1,102) patients. 1 of the reviews found 15 RCTs [5], while none of the other reviews found any RCTs. After reviewing the included studies, we found that 8 of the references could not be found from PubMed or Google Scholar (Li 2017, Shi 2018a, Shi 2018b, Zhu 2018, Wang 2018, Li 2018a, Li 2018b, Liu 2018), 6 studies were clearly reported as retrospective studies (Meng 2013, Nielsen 2008, Peterson 2010, Townshend 2013, Quayle 2018, Woo 2019), and 1 was stated to be an RCT (Hou 2017), but the full-text version of the article was not found [5]. The primary outcomes varied across all the meta-analyses, including fusion rate, complication rate, and PROMs. None of the studies specified predetermined primary time points.

2 of the reviews did not assess the risk of bias in the included studies, while 1 study used the RoB I tool, 1 used the RoB II tool, and 3 studies applied the Newcastle–Ottawa Scale (NOS) for observational studies. 1 study applied the GRADE approach appropriately and drew conclusions per outcome based on the certainty of evidence [43]. 1 of the studies mentioned using the GRADE approach to assess the certainty of the evidence but the reporting was not transparent and it is unclear which factors impacted the certainty ratings [5]. Certainty was reported as a single paragraph mentioning that the certainty was either “medium” (rather than “moderate,” as defined by GRADE) or “low.” The most common reasons for downgrading were “unclear random method” and “calculation of the optimal sample size.” However, these factors alone are not reasons for downgrading the certainty level. No additional reasons were provided, making the application of the GRADE approach non-reproducible. The overall confidence based on the AMSTAR 2 criteria was Critically Low (flaws in 5–16 out of 16) (see Table 1). Flaws were present in 4–7 out of 7 critical items.

All reviews concluded that arthroscopic-assisted surgery was superior to open surgery, none acknowledging that the evidence was drawn from observational studies and none performed analysis based on RCTs (see Table 2). 1 study found that arthroscopic-assisted surgery is associated with higher complication rates, yet still concluded that the surgeons should have “good arthroscopic skills” when performing these procedures [41].

### Arthroscopic-assisted reduction for ankle fractures

7 reviews investigated the effect of arthroscopic surgery for ankle fractures [2,3,7,27,34,35,44], all comparing arthroscopy-assisted and open reduction and internal fixation for ankle fractures. 4 of the reviews performed meta-analysis. The reviews were published between 2015 and 2025. They included a median of 10 (range 4–18) studies and median of 597 (range 188–861) patients. 5 reviews included RCTs, of which none included only RCTs. The primary outcomes were PROMs in all studies.

2 of the reviews did not assess the risk of bias in the included studies, while 2 studies used the RoB II and 1 ROB I tools, 1 applied NOS for observational studies, and 1 used modified Coleman Methodology Score (CMS). None of the studies employed the GRADE approach to assess the certainty of the evidence.

The overall confidence based on the AMSTAR 2 criteria was Critically Low (flaws in 9–16 out of 16) for all reviews (see Table 1). Flaws were present in 5–7 out of 7 critical items.

The conclusions varied between arthroscopic-assisted surgery being superior and comparable to open surgery, none acknowledging that the evidence was drawn from observational studies and none performed analysis based on RCTs (see Table 2).

### Debridement or autologous chondrocyte implantation for osteochondral lesions

4 reviews investigated the effect of arthroscopic surgery for osteochondral defects including different interventions, such as debridement only, debridement and drilling, debridement and curettage, microfractures, internal fixation of the loose bodies, and arthroscopic matrix induced autologous chondrocyte implantation (MACI) [2,28,29,33]. None of the reviews performed meta-analysis. The reviews were published between 2015 and 2021. They included a median of 19 (range 4–52) studies and median of 343 (range 189–1,361) patients. 3 reviews included RCTs, of which none included only RCTs. The primary outcomes were PROMs in all studies.

1 of the reviews did not assess the risk of bias in the included studies, while 1 study used the NOS, 1 the modified CMS, and 1 applied the ROBIS tool. None of the studies employed the GRADE approach to assess the certainty of the evidence.

The overall confidence, based on the AMSTAR 2 criteria, was Critically Low for all included reviews (flaws in 12–16 out of 16). Flaws were present in 6–7 out of 7 critical items.

The conclusions were generally cautious, with no review asserting the efficacy of any surgical approach and most stating that the available evidence is insufficient to support 1 type of surgery over another, none acknowledging that the evidence was drawn from observational studies and none performed analysis based on RCTs (see Table 2).

### AMSTAR 2 criteria

Included reviews revealed several critical flaws in review methods, as assessed by the AMSTAR 2 criteria (see Table 1). The most common issues were related to assessing study protocol (item 2), search strategy (item 4), and risk of bias assessment (item 9), none of which were conducted properly in any of the studies. Among these, items 2, 4, and 9 are classified as critical items according to the AMSTAR 2 criteria. Additionally, 27 studies did not specify reasons for excluding studies (Item 7) and 20 did not specify reasons for included non-randomized studies (Item 3). At best, 1 of the reviews met up to 11 out of 16 of the AMSTAR 2 criteria [43].

## Discussion

In this umbrella review of systematic reviews on ankle arthroscopy, we found no evidence supporting the efficacy of ankle arthroscopic surgery in chronic lateral ankle instability, ankle fractures, talar osteochondral defects, ankle osteoarthritis, impingements, removing loose bodies, septic arthritis, arthrofibrosis, or synovitis. Specifically, no studies compared arthroscopic surgery with placebo or no treatment, nor did we identify evidence demonstrating its superiority over nonoperative management. Consequently, the perceived efficacy of these procedures is primarily based on post-surgical improvements, which does not establish causality—patients may improve despite, rather than because of, surgery. Therefore, no causal conclusions can be drawn from the existing body of literature and further reviews based on the same methodologically flawed primary data will constitute a waste of research. Furthermore, the quality of systematic reviews concluding benefits of arthroscopic surgery over open procedures was poor: methods were inadequate or transparency lacking, and conclusions rarely reflected the uncertainties in the available evidence.

Treatment practices in modern medicine rely on data from high-quality RCTs, which can be synthesized in meta-analyses that provide a comprehensive evaluation of both benefits and harms. A well-conducted interventional systematic review and meta-analysis should go beyond pooling data; it must also assess the certainty of the best available estimates of treatment effect and communicate shortcomings of the evidence clearly in the conclusion [10,22]. None of the reviews in the present study managed to account for all factors that impact the certainty of evidence, and most failed on multiple aspects [12].

Conclusions from systematic reviews need to be rigorous because they typically contribute to the treatment guidelines [22]. Including data from flawed studies is not necessarily a critical problem per se if the conclusions reflect the uncertainties and the review highlights the current key evidence gaps to guide future research. The problem arises when firm and biased conclusions are drawn based on biased data. This can mislead readers and steer guidelines in the wrong direction, potentially causing harm to patients, and was often found in our study [11].

Most of the included reviews supported the use of ankle arthroscopy, despite the absence of studies directly comparing it with nonoperative treatment. These selectively interpreted conclusions highlight a second concerning practice referred to as spin [50,51]. Even when the data does not demonstrate any benefits over no surgery, or the benefits are clearly negligible compared with open procedures, the reviews may still claim benefits for arthroscopy. This problem has been recognized outside these reviews [50-57], and is commonly observed in orthopedic research as well [58-60]. The presence of spin can be either intentional or unintentional, driven by factors such as funding-related reasons or the need to enhance the manuscript’s likelihood of publication [60].

Our study revealed first that greater efforts should be made to compare the benefits of ankle arthroscopy against placebo or nonoperative controls through randomized controlled trials, as this is the only reliable way to evaluate the true effect of any intervention. Second, systematic reviews and meta-analyses should adhere to established guidelines such as PRISMA and apply the GRADE approach when synthesizing evidence [10,14,61]. This would enhance methodological quality and lead to more robust conclusions, thereby improving their suitability for clinical decision-making. Third, journal editors and peer reviewers should require transparent reporting and ensure that conclusions accurately reflect the uncertainty of the underlying evidence.

### Strengths

We used a systematic and transparent approach to evaluate and report methodological issues across the included studies. The umbrella review design offers a unique advantage by providing a comprehensive synthesis of evidence from multiple systematic reviews, particularly those commonly used to inform clinical decision-making. Such a broad, high-level perspective is not achievable with traditional review designs. Additionally, the review protocol was prospectively registered, and any deviations from the original plan were clearly documented and updated in the registry to ensure transparency.

### Limitations

Due to non-transparent reporting of the included studies, we were sometimes unable to assess whether proper methods were used or to explain how studies arrived at the conclusions they did. As an umbrella review, this study was not aimed at estimating the effects of studied interventions but rather at summarizing evidence at a higher level than RCTs, whether reviews are reliable for clinical guidelines, and to identify key evidence gaps.

### Conclusions

The efficacy of ankle arthroscopic procedures remains based solely on observational evidence. Given the critically low methodological quality of existing reviews, conclusions suggesting benefits of ankle arthroscopy, particularly over open procedures, are unreliable and insufficient to inform clinical recommendations. RCTs comparing ankle arthroscopy with nonoperative treatments or sham surgery are urgently needed.

*In perspective,* to advance the field, we propose that: (i) greater efforts be made to compare the benefits of ankle arthroscopy against placebo or nonoperative controls with RCT; (ii) systematic reviews and meta-analyses adhere to established guidelines (PRISMA) and apply the GRADE approach to synthesize the evidence. Furthermore, journals should require transparent reporting and ensure that conclusions appropriately reflect the uncertainty of the evidence.

### Supplementary data

Appendices 1–4 are available as supplementary data on the article page, doi: 10.2340/17453674.2025.44330

## Supplementary Material



## Notes

VPo, AL, VM, and TK conceived the study idea. VPo conducted the literature searches. VPa, JL, and VPo screened the articles and extracted the data. VPo wrote the initial draft of the manuscript. All authors reviewed and revised the draft, and approved the final manuscript for publication.

Handling co-editors: Bart A. Swierstra and Robin Christensen

*Acta* thanks Stig Brorson and Rudolf W Poolman for help with peer review of this manuscript.
