# Supplementary material for: Uncertainty and risk of misleading conclusions: an umbrella review of the quality of the evidence for ankle arthroscopy
Source: Acta Orthop. 2025 Jul 25;96:574–83. doi: 10.2340/17453674.2025.44330 (PMC12292010; doi:10.2340/17453674.2025.44330)
Supplement: Supplementary file 1 [file ActaO-96-44330-s1.pdf]

Supplementary data

Appendix 1. Search strategy for each database. No filters or limits were applied.

MEDLINE
(("ankle fracture*" OR "ankle instability" OR "osteochondral lesion*" OR "osteochondritis dissecans" OR
"ankle arthrodesis" OR "ankle fusion" OR "ankle injur*" OR "ankle condition*") AND
("ankle arthroscop*" OR ("arthroscop*" AND "ankle")) AND
("systematic review" OR "meta-analys*" OR "systematic overview"))

CENTRAL
(("ankle fracture*" OR "ankle instability" OR "osteochondral lesion*" OR "osteochondritis dissecans" OR
"ankle arthrodesis" OR "ankle fusion" OR "ankle injur*" OR "ankle condition*")
AND
("ankle arthroscop*" OR ("arthroscop*" AND "ankle"))
AND
("systematic review" OR "meta-analys*" OR "systematic overview"))

Embase
(('ankle fracture*' OR 'ankle instability' OR 'osteochondral lesion*' OR 'osteochondritis dissecans' OR
'ankle arthrodesis' OR 'ankle fusion' OR 'ankle injur*' OR 'ankle condition*'):ti,ab)
AND
(('ankle arthroscop*' OR ('arthroscop*' AND 'ankle')):ti,ab)
AND
(('systematic review' OR 'meta-analys*' OR 'systematic overview'):ti,ab)

Appendix 2. Excluded full-text studies

Study ID	Citation	Exclusion reason
Tornberg 2025	Tornberg, H., Hartman, H. C., Fine-Lease, P., & Gianakos, A. (2025). Podium Presentation Title: Arthroscopic Management of Talus Fracture: A Review of the Clinical Outcomes and Surgical Technique. *Arthroscopy*, *41*(2), e17-e18.	Awaiting classification
Jagtenberg 2021	Jagtenberg EM, Kalmet PHS, de Krom MAP, Hermus JPS, Seelen HAM, Poeze M. Effectiveness of surgical treatments on healing of cartilage and function level in patients with osteochondral lesions of the tibial plafond: A systematic review. J Orthop. 2021 Aug 17;27:34-40. doi: 10.1016/j.jor.2021.08.011.	Wrong intervention
Huang 2023	Huang M, Li Y, Liao C, Lai Q, Peng J, Guo N. Microfracture surgery combined with platelet-rich plasma injection in treating osteochondral lesions of talus: A system review and update meta analysis. Foot Ankle Surg. 2024 Jan;30(1):21-26. doi: 10.1016/j.fas.2023.09.004.	Wrong intervention

Appendix 3. Search strategy for each database for the first search on December 16, 2024. The search was conducted to include original RCTs comparing ankle arthroscopy with placebo, sham surgery or nonoperative treatment on December 22nd 2024. No filters or limits were applied.

MEDLINE
1. Ankle/
2. Ankle Injuries/
3. Ankle pain.tw
4. Or/1-3
5. Arthroscopy/
6. Arthroscop*.ti,ab.
7. 5 or 6
8. randomized controlled trial.pt.
9. controlled clinical trial.pt.
10. randomized.ab.
11. placebo.ab.
12. drug therapy.fs.
13. randomly.ab.
14. trial.ab.
15. groups.ab.
16. or/8-15
17. exp animals/ not humans.sh.
18. 16 not 17
19. And/4,7,18
20. Meta-Analysis.pt.
21. Systematic Review.pt.
22. meta-analysis.tw OR systematic review.tw
23. Or/20-22
24. Or/19,23And/4,7,18

CENTRAL
1. MeSH descriptor: [Ankle]
2. MeSH descriptor: [Ankle Injuries]
3. Ankle pain
4. MeSH descriptor: [Talus]
5. Calcaneus
6. Navicular
7. Cuboid
8. Cuneiform*
9. Distal tibia
10. Talar dome
11. Ankle joint
12. Subtalar joint
13. Tibiotalar

14. Syndesmosis
15. Posterior malleolus
16. Lateral malleolus
17. Medial malleolus
18. Deltoid ligament
19. ATFL (anterior talofibular ligament)
20. CFL (calcaneofibular ligament)
21. PTFL (posterior talofibular ligament)
22. #1 or #2 or #3 or #4 or #5 or #6 or #7 or #8 or #9 or #10 or #11 or #12 or #13 or #14 or #15 or #16 or #17 or #18 or #19 or #20 or #21
23. MeSH descriptor: [Arthroscopy]
24. #22 and #23

Embase

1. Ankle/
2. Ankle Injuries/
3. Ankle pain.tw
4. Or/1-3
5. Arthroscopy/
6. Arthroscop*.ti,ab.
7. 5 or 6
8. 4 and 7
9. Clinical Trial/
10. Randomized Controlled Trial/
11. exp randomization/
12. Single Blind Procedure/
13. Double Blind Procedure/
14. Crossover Procedure/
15. Placebo/
16. Randomized controlled trial$.tw.
17. Rct.tw.
18. random allocation.tw.
19. randomly allocated.tw.
20. allocated randomly.tw.
21. (allocated adj2 random).tw.
22. Single blind$.tw.
23. Double blind$.tw.
24. ((treble or triple) adj blind$).ti.
25. placebo$.tw.
26. prospective study/
27. or/9-26
28. 8 and 27
29. Meta-Analysis.pt.
30. Systematic Review.pt.
31. meta-analysis.tw OR systematic review.tw
32. Or/29-31
33. Or/28,32

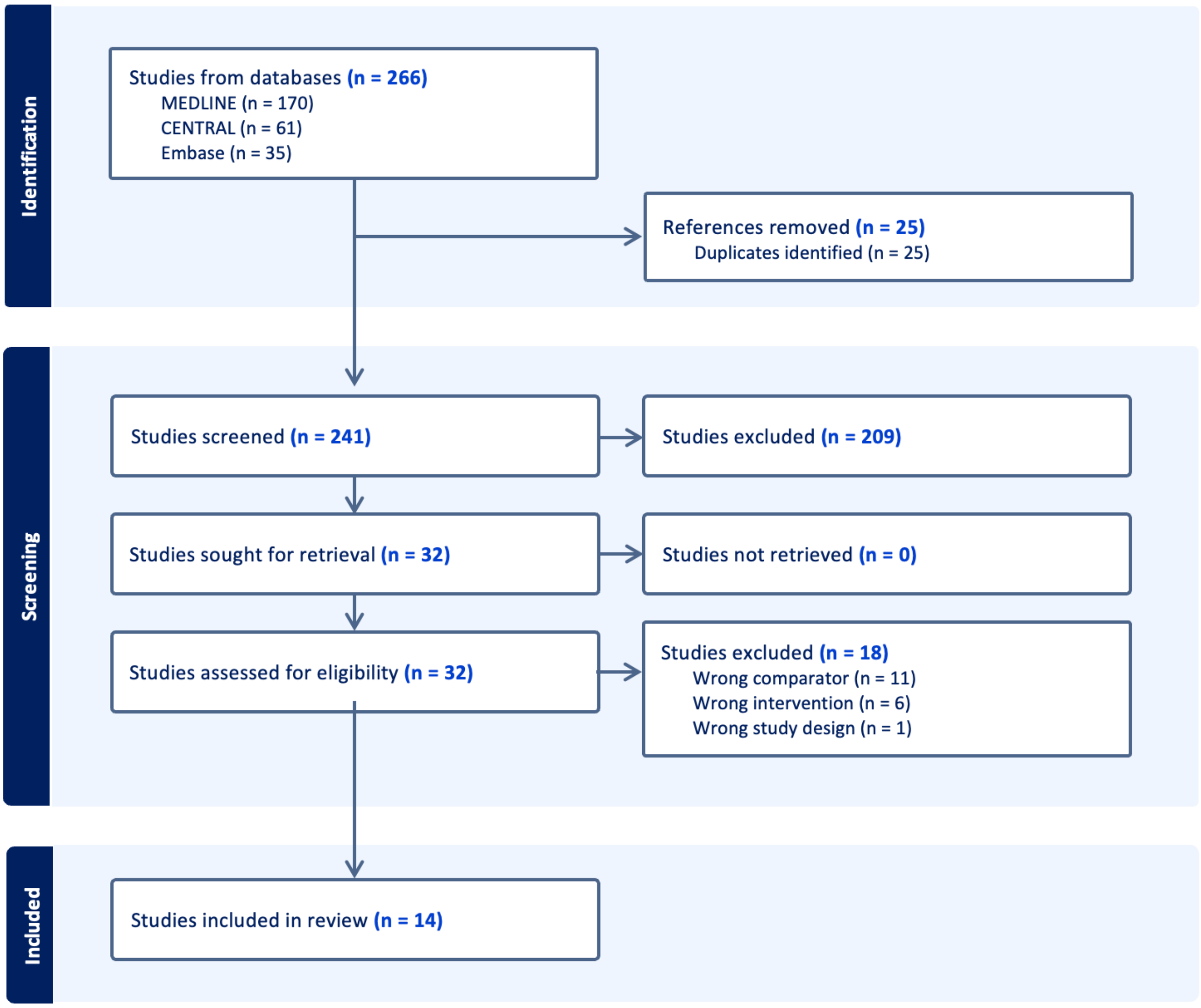

Appendix 4. Flow chart of the review process for the first search on December 16, 2024. Original articles were not found, yet we included 14 eligible systematic reviews for this umbrella review.
